# Dairy Fat Consumption and the Risk of Metabolic Syndrome: An Examination of the Saturated Fatty Acids in Dairy

**DOI:** 10.3390/nu11092200

**Published:** 2019-09-12

**Authors:** Allison L. Unger, Moises Torres-Gonzalez, Jana Kraft

**Affiliations:** 1 Department of Animal and Veterinary Sciences, The University of Vermont, Burlington, VT 05405, USA, Allison.Unger@uvm.edu; 2 National Dairy Council, Rosemont, IL 60018, USA; 3 Department of Medicine, Division of Endocrinology, Metabolism and Diabetes, The University of Vermont, Colchester, VT 05446, USA; Jana.Kraft@uvm.edu

**Keywords:** abdominal obesity, branched-chain fatty acids, cardiometabolic, dyslipidemia, hyperglycemia, hypertension, insulin resistance, medium-chain fatty acids, odd-chain fatty acids, short-chain fatty acids

## Abstract

Lifestyle is a key modifiable risk factor involved in the manifestation of metabolic syndrome and, in particular, diet plays a pivotal role in its prevention and development. Current dietary guidelines discourage the consumption of saturated fat and dietary sources rich in saturated fat, such as dairy products, despite data suggesting that full-fat dairy consumption is protective against metabolic syndrome. This narrative review assessed the recent epidemiological and clinical research that examined the consumption of dairy-derived saturated fatty acids (SFA) on metabolic syndrome risk. In addition, this review evaluated studies of individual SFA to gain insight into the potential mechanisms at play with intake of a diet enriched with these dairy-derived fatty acids. This work underscores that SFA are a heterogenous class of fatty acids that can differ considerably in their biological activity within the body depending on their length and specific chemical structure. In summary, previous work on the impact of dairy-derived SFA consumption on disease risk suggests that there is currently insufficient evidence to support current dietary guidelines which consolidate all dietary SFA into a single group of nutrients whose consumption should be reduced, regardless of dietary source, food matrix, and composition.

## 1. Introduction

Metabolic syndrome (MetS) is a serious health condition characterized as a minimum of three of the following physiological components: hyperglycemia, abdominal obesity, atherogenic dyslipidemia (low HDL-cholesterol and high plasma triacylglycerols), and hypertension [1]. While not a disease *per se*, MetS predisposes an individual to developing a wide range of other diseases, including type 2 diabetes, cardiovascular disease (CVD), and strokes [2]. MetS has become an international health crisis and currently afflicts approximately 25% of the global population [3]. The risk of MetS incidence varies with age [4], sex [5], and genetics [2,5] of an individual, yet lifestyle pattern is recognized as the leading modifiable factor in its development [5]. Specifically, dietary intervention remains the principal recommendation for the prevention and management of MetS [5].

Currently, the U.S. and international dietary guidelines advocate a limited inclusion of saturated fat as part of an optimal strategy for prevention of cardiometabolic diseases [6,7,8,9]. By extension, these dietary guidelines explicitly discourage the consumption of foods that are rich in saturated fat, such as full-fat and reduced-fat dairy products. These recommendations were originally based on studies performed in the early 20th century that indicated a positive association between dietary saturated fat content and CVD [10] while, importantly, more recent studies assessing the impact of saturated fat intake on metabolic health are inconclusive with widely variable results [11]. A notable example is a recent prospective study which found that intake of total saturated fat was positively associated with mortality risk, while consumption of medium- and odd-chain saturated fatty acids (SFA) was negatively associated with mortality risk [12]. Clearly, these discrepancies are likely due, in part, to the heterogeneity of SFA. Depending on the dietary source, SFA vary significantly in carbon length and structure [13,14]. Hence, the importance of dietary SFA chemical heterogeneity is more complex than previously recognized, and the physiological impact of dietary SFA depends not only on the dietary source and food matrix, but also the SFA type(s) and composition [12,15,16]. As part of the scientific debate surrounding the assumption that saturated fat consumption accounts for an increased incidence of cardiometabolic diseases, the role of dairy consumption as a component of a healthy diet remains a central theme of discussion. While SFA are quantitatively the predominant class of fatty acids (FA) in dairy fat, it is rather unique as it is also comprised of a wide and complex variety of SFA, including short-, medium-, long-, odd-, and branched-chain FA [17]. Epidemiological studies have shown that habitual consumption of dairy products may reduce risk of metabolic diseases [18,19], and in particular, that dairy fat may have protective properties to attenuate development of MetS in adult and elderly populations [20,21]. Therefore, it is likely that the diverse array of SFA constituents within full-fat dairy foods contributes to favorably modulating cardiometabolic health. Whereas considerable research effort has been devoted to health effects of dietary saturated fat in general, rather little attention has been paid to the effects of dairy-derived SFA on the risk of MetS.

The overarching goal of this narrative review is to evaluate the current evidence on the effects of dairy-derived SFA consumption on MetS risk and assess whether SFA type and composition influences cardiometabolic risk. The first objective was to review recent literature that focused on the effects of dairy-derived SFA on MetS and any of its individual risk factors (i.e., hyperglycemia, (abdominal) obesity, dyslipidemia, or hypertension). The second objective was to identify the potential mechanisms involved through the discussion of mechanistic studies that examined the effects of individual SFA found in dairy. Finally, this review discusses the gaps and limitations present in the literature and assesses whether there is sufficient evidence to support current national dietary guidelines which regard dietary SFA as a single group of nutrients.

## 2. Background of SFA Found in Dairy

Dairy fat is composed of more than 400 different identified FA and FA derivatives, but only about 15 FA represent quantities greater than 1% of total FA [22]. SFA are quantitatively the predominant FA class in milk fat, accounting for a range approximately of 64–73% of total FA (Table 1) [17,23,24], or 5 g per serving of whole milk [25]. However, unlike other animal-derived fats, milk fat’s SFA uniquely consists of SFA with varying carbon chain length ranging from 4 to 24 carbon atoms [22]. The majority of SFA are long-chain FA (14:0–21:0, 58% of total FA; Table 1), primarily derived from the diet or tissue stores of the ruminant itself, with palmitic acid (16:0) being the prominent SFA (35% of total FA) followed by stearic acid (18:0) and myristic acid (14:0) (9 and 12% of total FA, respectively (Table 1)). The content of medium-chain SFA (7:0–13:0) and short-chain SFA (4:0–6:0) in milk fat, both synthesized in the mammary gland via de novo lipogenesis [17], is about 8% and 5% of total FA, respectively (Table 1).

Branched-chain FA (BCFA) are distinctive, well-established constituents of milk fat and have their origin in the rumen where they are synthesized de novo or metabolized from phytol by rumen microorganisms [27]. Accounting for almost 2% of milk (Table 1), BCFA are commonly SFA substituted with one (mono-) or more (di-/poly-) methyl branch(es) on the carbon chain. Typically, BCFA possess either an *iso* structure where the FA has the branch point on the penultimate carbon atom (*n*-2 carbon atom) or an *anteiso* structure where the branch point is located on the antepenultimate carbon atom (two from the end, *n*-3 carbon atom). *Iso-* and *anteiso*-mono-methyl BCFA with chain lengths from 13 to 17 carbon atoms are quantitatively the most abundant BCFA in milk fat [28]. Milk fat also contains a minor amount (3% of total FA; Table 1) of bacterial-derived odd-chain FA (OCFA), primarily comprised of pentadecanoic acid (15:0) and heptadecanoic acid (17:0), each about 1% of total FA (Table 1), respectively. These two OCFA are measured in plasma, serum phospholipids, erythrocyte membranes, and adipose tissue, and are used as biomarkers of dairy fat intake [29,30].

## 3. Dairy-Derived SFA Intake Recommendations in Dietary Guidelines

**Figure 1:**
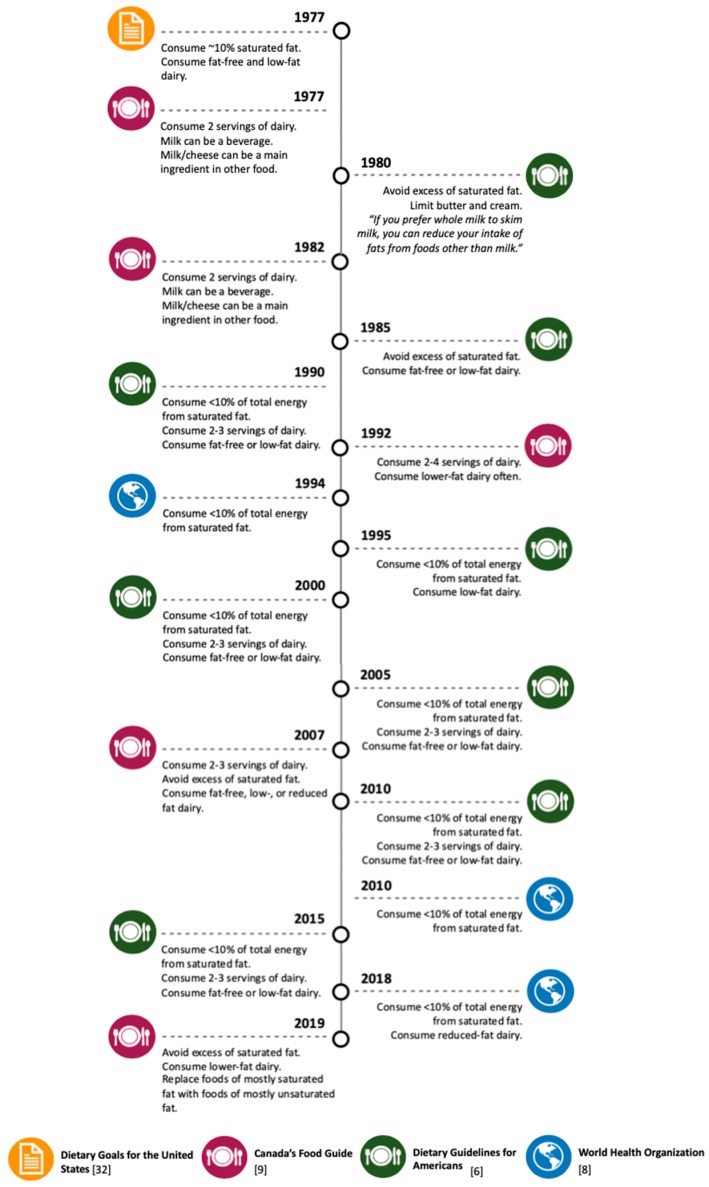
Timeline of the evolvement of dietary recommendations of total, saturated, and dairy- derived fat intake for the United States, Canada, and world health organization.

The “diet-heart hypothesis”, which postulates that diets high in saturated fat cause CVD [31], is a paradigm that has influenced U.S. national dietary guidelines since their establishment (Figure 1) [6]. Looking back at the 1977 dietary goals for the U.S. [32], dairy fat has historically been targeted because of its high content of saturated fat, prompting public health authorities to caution against consuming full-fat dairy products (Figure 1). Since then, U.S. dietary guidelines have remained steadfast in their recommendation to substitute fat-free or low-fat dairy for higher-fat dairy products [6]. Mounting evidence, however, supports that the “diet-heart hypothesis” is an oversimplification of dietary saturated fat and that health effects of saturated fat are considerably dependent upon other important factors, such as food source and matrix [33], the overall dietary pattern of an individual, and the health status of the individual [34,35]. Paradoxically, despite a more thorough understanding of the heterogenous health effects of dietary SFA, dietary guidelines outside of the U.S. also continue to discourage full-fat dairy products (Figure 1). For example, Canada’s recently released 2019 food guide discourages the consumption of higher-fat containing dairy products [9]. Furthermore, in 2018, the World Health Organization recommended the consumption of reduced-fat dairy for the first time [8].

## 4. Epidemiological and Clinical Studies: The Effects of Dairy-Derived SFA on MetS or One of Its Four Risk Factors

### 4.1. Methods

The Ovid MEDLINE database was searched, combining the three following search term categories with AND: (1) “exp Cholesterol/” OR “Dyslipidemias/” OR “Obesity/” OR “Obesity, Abdominal/” OR “exp Hyperglycemia/” OR “exp Insulin Resistance/” OR “Hypertension/” OR “Cardiometabolic.mp.”, (2) “exp Dairy Products/”, and (3) “exp Fatty Acids/” OR “Saturated Fat.mp.” OR “Saturated Fatty Acids.mp.”. Search results were limited to publications written in the English language and published over the past ten years between December 2008 and December 2018. Studies were excluded if they did not identify dairy-derived SFA or did not assess an outcome of MetS or any of its individual physiological components. The references of the included articles, as well as any relevant meta-analyses or review articles, were manually searched to identify other eligible studies.

### 4.2. MetS

Observational research supports that dairy fat intake is protective against MetS development [20,21]. A complete description of the observational and clinical studies discussed in this portion of the review can be found in Table S1. Two cross-sectional studies were identified, which evaluated the influence of consumption of SFA within dairy on the risk of MetS (Table 2) [21,36]. In a cohort of 9835 middle-aged and elderly men and women in Brazil, Drehmer et al. [21] observed a graded, inverse, and independent relationship of total and full-fat dairy consumption with the MetScore (a score to estimate MetS risk of a participant). This study also revealed, by adjusting for dairy SFA intake in their model, that SFA in dairy may be critical nutrients in the relationship between full-fat dairy intake and MetS prevention. In a small cohort study from Spain, 427 elderly participants with high risk of CVD were enrolled to examine the relationship between plasma FA and MetS prevalence. When subjects were segregated into quartiles based on the proportion of 17:0 in their plasma, the second quartile, but not the third or fourth quartile, was associated a decreased risk of MetS [36]. Taken together, cross-sectional studies indicate that dairy FA intake may be protective or neutral against development of MetS.

### 4.3. Hyperglycemia

Large-scale cross-sectional studies provide evidence that dairy fat consumption plays a role in glucose homeostasis [37,38]. Since the 1980s, it has been known that insulin resistance is a critical risk factor of MetS development [39]. Insulin resistance is likely established in an individual for several years before hyperglycemia develops [40], hence, indices of glucose tolerance, insulin sensitivity, and β-cell function are valid measurements to assess risk of metabolic diseases, such as type 2 diabetes and MetS. A study with a multiethnic population of adult men and women free of type 2 diabetes from the U.S. observed that serum concentrations of 15:0 were positively associated with log SI (insulin sensitivity index) and log DI (disposition index), both proxies for insulin sensitivity and β-cell function, respectively (Table 2) [41]. As FA composition in adipose tissue reflects long-term dietary intake, Iggman et al. [42] investigated the FA profile in adipose tissue in a cohort of 1221 elderly Swedish men and found that 17:0, but not 15:0, was positively correlated with insulin sensitivity indices (Table 2). In a recent cohort study of 5675 non-diabetic, middle-aged Dutch participants, Wanders et al. [43] demonstrated that the consumption of SFA from dairy products was inversely associated with fasting insulin concentrations and homeostatic model assessment of β-cell function (HOMA-B) in overweight adults (Table 2). Drehmer et al. [44] observed a beneficial relationship between total dairy intake and glycemia measurements in adult and elderly men and women, and found that consumption of 14:0 was likely involved in this mechanism. Thus, results from cross-sectional studies across various geographic regions support an inverse relationship between dairy-derived SFA biomarkers and the risk of hyperglycemia and insulin resistance (Table 2) [41,42,43,44,45].

In a feeding trial in Denmark, abdominally overweight participants were provided foods totaling 63 g/d of butter consisting of either a high (8.5 g/day) or slightly lower (6.9 g/day) amounts of medium-chain SFA (6:0–12:0; Table 3) [46,47]. After 12 weeks, neither group differed from one another in measurements of insulin sensitivity (HOMA of insulin resistance (HOMA-IR) and Matsuda Index), fasting glucose and insulin, or hemoglobin A1C. Similarly, Werner et al. [48] investigated Danish adults for the effect of butter consumption (39 g/d) containing two different proportions of SFA (59% and 64% SFA) for 12 weeks on metabolic risk factors and reported no changes in glucose or insulin measurements between groups (Table 3). A multicenter (Finland, Norway, and Sweden) study, whereby overweight men and women with risk factors of MetS consumed either 3–5 servings of dairy products daily, or maintained their habitual diet (control group) for six months, observed improved insulin sensitivity (via HOMA-IR) in the dairy group [49] (Table 3). However, no significant correlation between insulin sensitivity and a biomarker of dairy SFA intake (15:0; % of total serum cholesterol esters) was detected. In addition, findings from other clinical trials that compared the consumption of dairy-derived vs. plant-derived FA showed no differences in glucose homeostasis parameters (Table 3) [50,51]. Overall, results from clinical studies examining direct measures of glucose homeostasis with the dairy-derived SFA consumption are not conclusive and generally neutral.

### 4.4. Obesity

Abdominal obesity is the most common risk factor observed in patients with MetS [52]. Although it is poorly understood, accumulation of fat in the abdominal region, specifically visceral fat, poses a greater risk for development of cardiometabolic diseases than in other fat depots within the body [53,54,55]. Overall, literature thus far supports that high-fat dairy intake is neutral or protective against obesity development [37,38]. This review considered all epidemiological or clinical research that examined the effects of dairy SFA on parameters of obesity, rather than abdominal obesity specifically, due to the scarcity of studies available. In one clinical trial in Swedish, abdominally overweight adults examined the difference between a diet rich in SFA from butter vs. a diet rich in *n*-6 PUFA from sunflower oil and margarine consumed over ten weeks (Table 3) [50]. Body weight, waist circumference, and total fat mass remained unchanged in the SFA group compared to the *n*-6 PUFA group, although there was a small decrease in visceral/subcutaneous adipose tissue ratio in the *n*-6 PUFA vs. the SFA group. In other clinical trials, ranging in intervention duration from three to six months, similar neutral findings were observed (Table 3) [48,49,56]. Bohl et al. [47] found no differences in body weight of abdominally overweight subjects consuming either butter low in medium-chain SFA (6.9 g/day) or high (8.5 g/day) in medium-chain SFA, but in a follow-up analysis, the latter coincided with increased lean mass and decreased body fat percentage (Table 3) [46]. In general, clinical trials suggest that an increased consumption of dairy-derived SFA resulted either in neutral [47,48,49,50,51,56,57] or favorable [46,58] changes in body weight and body composition (Table 3).

### 4.5. Atherogenic Dyslipidemia

Abnormal blood lipid levels that are characteristic of MetS, such as low HDL-cholesterol and high plasma triacylglycerols, are an important indicator for cardiometabolic risk, as they are inextricably linked to poor energy homeostasis, insulin resistance, and ectopic and visceral fat accumulation [59]. Research has shown that dairy fat intake is associated with favorable blood lipids, including higher HDL cholesterol and lower triacylglycerol levels [37,38]. Generally, clinical trials that compared the relative amount of SFA within the same dairy food matrix [46,47,48,56,60] (e.g., butter with two different FA profiles) did not find any alterations in blood lipids among treatment groups (Table 3). One exception was a randomized, controlled trial (RCT) that found inconsistent effects on cholesterol levels of participants consuming a diet with 55 g/d of dairy fat comprising either 72%, 63%, or 57% SFA in French adults (Table 3) [61]. After three weeks, those consuming the 63% SFA, yet not 57% SFA, dairy fat had a lower LDL/HDL cholesterol ratio than those consuming the 72% SFA butter. Pintus et al. [57] tested the effect of cheese intake quantity (45 vs. 90 g/d) and SFA composition (59% vs. 46% SFA) on blood lipids (Table 3). There were no differences in blood lipid measurements when hypercholesterolemic participants consumed 45 g/d of cheese, regardless of the proportion of SFA, but 90 g/d of cheese containing 59% SFA resulted in increased HDL cholesterol. Notably, while the majority of studies compared SFA composition within dairy products, some studies were designed to compare the health effects of dairy-derived vs. plant-derived FA and reported variable results (Table 3) [50,51]. Therefore, variable study designs among RCTs [46,47,48,49,50,51,56,57,60,61] make it challenging to draw comparisons and conclusions, and to ascertain a genuine relationship between dairy SFA consumption and dyslipidemia (Table 3).

### 4.6. Hypertension

Hypertension impairs cardiovascular function and significantly predisposes an individual to CVD. Relatively less attention has been given to hypertension in the context of MetS, however, and the mechanisms by which elevated blood pressure and the other three main pathophysiological components of MetS are related are not well defined [3]. While there is limited research available that evaluates the relationship between full-fat dairy intake and hypertension, results from a recent randomized, controlled trial suggest a neutral effect of high-fat dairy consumption on blood pressure [63]. While only a few studies assessing hypertension or its risk factors were covered under this review, the limited number of relevant studies available also does not support a role for dairy-derived SFA in blood pressure modulation (Table 3) [46,49].

### 4.7. Limitations of Epidemiological and Clinical Studies Examining Dairy-Derived SFA Consumption and MetS

Epidemiological and clinical studies that assess the role of dairy-derived SFA on MetS risk have significant limitations to be considered when interpreting the findings. For example, three studies relied on estimated dairy SFA intake that was assessed through a one-time food frequency questionnaire [21,43,44], in some cases covering the previous twelve months [21,44]. Consequently, the accuracy of self-reported data must be considered with caution. Another study correlated odds ratios of MetS with FA biomarkers analyzed from plasma [36]; however, because plasma contains primarily FA from triacylglycerols, this analysis does not reflect long-term dietary intake, but rather the previous meal consumed [63]. Additionally, within any cross-sectional study, it cannot be discounted that the apparent association between dairy-derived SFA and MetS is due, at least in part, to other nutrients within dairy.

While randomized, clinical trials have many advantages over cross-sectional studies (e.g., determination of causality), they depend on a properly controlled study design. Accordingly, a significant limitation in most of the clinical trials to date is that the study design did not control for the dietary intake of participants [46,47,48,49,50,56,57,58,60,61,62]. The heterogeneity of diets between subjects severely restricts the reliability of these data, particularly when the intervention depended on small dietary changes (e.g., approximately 1.6 g/d difference in FA intake [46,47,58,62]). Additionally, some studies examined dietary dairy fat vs. plant-derived oils with the goal of comparing health effects of SFA vs. PUFA, respectively. Yet, because these fats are sufficiently distinct from one another in taste and appearance, the ability of researchers to properly blind participants to a treatment is challenging, if not impossible [50,51]. Lastly, matrix differences between foods, and even within dairy products (e.g., milk vs. yogurt vs. cheese), may confound results, which might have impacted the findings. Some clinical trials controlled for food matrix by comparing one type of dairy product (e.g., butter) with differing proportions of SFA. However, similar to an inherent limitation of cross-sectional studies, it is difficult to parse whether observed physiological effects are due to a fluctuation of other nutrients, such as a decrease in MUFA and/or PUFA, rather than an increase in SFA.

A general limitation that is important to acknowledge is the reliance on biomarkers such as 15:0 and 17:0 to estimate dairy fat consumption. For example, 15:0 and 17:0 occur in all ruminant-derived products, such as meat [64], and other non-ruminant foods [65,66,67,68], and can also be synthesized endogenously [69]. Moreover, technical issues can also arise during the fatty acid analysis via gas-liquid chromatography; 15:0 and 17:0 might be incorrectly identified as well as overestimated due to coelution with other FA [70]. However, despite this potential limitation, epidemiological and clinical research to date has demonstrated that dairy fat intake, compared to other foods, has shown stronger and consistent correlations with 15:0 and 17:0 blood concentrations. Therefore, while more research is needed to determine whether other biomarkers of dairy fat intake can be utilized, 15:0 and 17:0 for now seem to be appropriate indicators of dairy fat consumption.

## 5. Potential Mechanisms Driving Differential Effects of SFA Found in Dairy on MetS Components

### 5.1. Short-Chain FA

Butyric acid (4:0; Figure 2a) is hallmarked as an inhibitor of histone deacetylase [71,72,73] and an agonist of specific G protein-coupled receptors [74,75]. Consuming three servings of whole milk translates into ~700 mg butyric acid/day of (Table 1). In a four-week pilot study, healthy lean males vs. obese males with MetS were given oral doses of 4 g butyrate/day. Notably, butyrate improved peripheral and insulin sensitivity in healthy lean males but not in the obese males [33]. In addition, butyrate caused modest changes in colonic bacterial composition, but changes were different depending on whether males were healthy and lean versus obese with MetS. Thus, this study suggests that butyrate influences insulin sensitivity, and implicates that these beneficial actions may be achieved in part through modification of the gut bacterial community structure. Using the same cohort, Cleophas et al. [34] performed ex vivo experiments with the participants’ peripheral blood mononuclear cells and found that butyrate treatment led to a reduction of the overall inflammatory phenotype of circulating monocytes.

Animal studies also support that butyrate supplementation of the diet can induce positive changes in whole body metabolism. For example, in rodents, butyrate provided with a high-fat (HF) diet has been shown to mitigate weight gain [76,77,78,79,80,81,82] and adiposity [76,78,79,80]. Butyrate has also been observed to enhance FA substrate oxidation [78,79,80], and in some cases, to increase total energy expenditure [78,80]. Moreover, a HF diet enriched in butyrate resulted in improved glucose metabolism [76,77,78,79,80,81], lower blood lipid levels [81,82], and atherosclerosis protection [83]. Two studies demonstrated that dietary butyrate increases uncoupling protein UCP-1 expression in brown adipose tissue, suggesting that butyrate may enhance thermogenesis [76,78]. Li et al. [76] additionally found that UCP-1 expression was attenuated when mice received a subdiaphragmatic vagotomy, suggesting that butyrate acts, in part, through the so-called gut–brain axis. Moreover, there is evidence that dietary butyrate acts via the gut by inducing intestinal hormone secretions of GLP-1, GIP, and PYY [77]. Recent evidence suggests that that dietary butyrate possesses a range of biological activities, including the capability to regulate energy metabolism via activation of AMP kinase in skeletal muscle [78], downregulate lipogenic pathways in the liver [73,80] and adipose tissue [80], improve β-cell function [81], reduce inflammation [34,82], and modify gut bacterial composition [33,76,82] and/or short-chain FA receptor expression [82]. Lastly, the role of butyrate on satiety has been of increased interest, however, whether or not dietary butyrate is involved in appetite reduction is controversial [76,77,79,80] and needs to be further examined.

Relatively limited work has investigated valeric (5:0) and caproic (6:0) acids. Recently, it has been found that dietary valerate has anti-inflammatory properties and, like butyric acid, has histone deacetylase inhibitor activity [84]. In addition, studies performed in chicken hepatocytes show that dietary caproate may beneficially modulate lipid metabolism [85,86].

### 5.2. Medium-Chain Fatty Acids

Caprylic acid (8:0; Figure 2b) is unique because it is the only FA known to be involved in the acylation of ghrelin, a post-translational modification that is required for the peptide hormone’s ability to stimulate hunger sensing and growth hormone release [87]. Caprylate may also influence energy homeostasis via downregulating mRNA transcription of enzymes of FA uptake and synthesis in adipocytes [88]. In chicken hepatocytes, caprylate has been shown to reduce secretion of apolipoprotein B, a component of low density cholesterol particles [86], reduce VLDL-cholesterol synthesis [89], and inhibit FA synthase activity, a key enzyme in de novo lipogenesis [85]. Caprylic acid has been documented to enhance glucose-stimulated insulin secretion [90], and two recent studies indicate that caprylic acid can induce this response in a dose-dependent manner [91,92]. Moreover, in mechanistic studies using a murine β-cell line (MIN6), these effects of caprylic acid on insulin secretion are likely mediated by the olfactory receptor OLFR15 via the phospholipase C-inositol triphosphate-dependent pathway [91,92]. Thus, research suggests that caprylic acid intake may beneficially influence glucose and energy homeostasis.

Like caprylic acid, research supports that capric acid (10:0) may also positively modulate lipid metabolism. In rodents, diets enriched with capric acid improved blood lipid profiles, including a reduction of total cholesterol and triacylglycerol [93,94]. Caprate has been found to transcriptionally regulate lipogenesis and reverse fat accumulation in steatotic hepatocytes in vitro [95]. Incubation of chicken hepatocytes with caprate has also shown a dose-dependent reduction of apolipoprotein B mRNA [86]. Notably, one study provided insight into a direct mechanism of fatty acid induced gene expression demonstrating capric acid binding to peroxisome proliferator-activated receptor γ, a key regulator of lipogenesis, in a unique binding pocket [93]. Lee et al. [96] investigated in vitro and in vivo effects of capric acid on intestinal health. When IPEC-J2 cells, a non-transformed porcine intestinal epithelial cell line, were treated with the immunosuppressant cyclophosphamide, the addition of capric acid attenuated inflammation and oxidative stress, and enhanced mRNA expression of proteins related to improved intestinal barrier function. More strikingly, when pigs fed diets enriched with capric acid were challenged with cyclophosphamide, a similar phenotype was observed. Capric acid may therefore contribute to overall reduced metabolic risk via effects on cholesterol metabolism and intestinal inflammatory status.

Potential mechanisms of lauric acid (12:0) are underrepresented in the literature. Consuming three servings of whole milk translates into ~800 mg lauric acid/day of (Table 1). In a small crossover RCT, eight patients with type 2 diabetes were given a prescribed breakfast and lunch each with an enteric-coated pellet containing 2.35 g lauric acid [97]. This study provided evidence that lauric acid may lower postprandial glucose levels through stimulation of GLP-1 release. In spontaneously hypertensive rats, lauric acid, administered intravenously, lowered heart rate and blood pressure [98]. In addition, treatment of THP-1 macrophages with lauric acid decreased expression of A disintegrin and metalloproteinase with thrombospondin motifs (ADAMTS) −1, −4, and −5, which are key enzymes in the development of atherosclerosis [99]. Thus, the limited body of evidence suggests that lauric acid may exert anti-hypertensive and anti-atherosclerotic properties.

### 5.3. Long-Chain FA

Myristic acid (14:0) is involved in the acetylation of at least 0.5% of total proteins [100]. In addition, myristic acid has been found to have the highest binding potency with the human G protein-coupled receptor 40 (GPR40), a major LCFA receptor expressed in enteroendocrine and β-cells, compared to 6:0–23:0 SFA in experimental conditions [101]. Human studies thus far have primarily focused on myristic acid’s unfavorable impact on blood lipid levels [102,103]. Recent work in mice showed that dietary supplementation with myristic acid enhances glucose tolerance and insulin sensitivity [104]. Further investigation in C2C12 murine myotubes demonstrated that these beneficial effects may be mediated by promoting glucose uptake via stabilization and therefore increased expression of diacylglycerol kinase in skeletal muscle [105,106]. There is additional evidence that myristic acid enhances incorporation of *n*-3 FA into tissues [107,108], however, this mechanism is still not well understood.

The covalent linkage of palmitic acid (16:0) or stearic acid (18:0; Figure 2c) to a protein, known as palmitoylation and stearoylation, respectively, is a common post-translational modification. Whereas palmitoylation is well-established as an essential process in the proper function of variety of proteins, no significance was ascribed for this process with stearic acid until 2015, when Senyilmaz et al. [109] demonstrated that stearoylation may play a critical role in mitochondrial function. In vitro studies in human and *Drosophila* cell cultures showed that post-translational attachment of stearic acid to transferrin receptor 1 protein inhibits c-Jun n-terminal kinase signaling and subsequent mitochondrial fragmentation. Furthermore, experiments in *Drosophila* revealed that dietary stearic acid increased mitochondrial fusion, thus preserving cellular oxygen consumption capacity. Extension of this work demonstrated that in healthy and diabetic adults inclusion of 24 g stearic acid into a breakfast meal also induced mitochondrial fusion [110]. It is important to note that three servings of whole milk per day provide approximately 2 g of stearic acid (Table 1). In rodent models, dietary stearic acid has been observed to lower total plasma cholesterol [111,112] and its absorption [112], increase fecal excretion of FFA [113], and mitigate HF diet-induced body weight and fat accumulation [113]. The neutral effects of stearic acid on cholesterol in humans has been well confirmed and thoroughly reviewed elsewhere [114]. Additionally, results from Cowles et al. [115] indicate that stearic acid may impact cholesterol metabolism via modulation of secondary bile acid composition. Stearic acid has also been implicated in modulating inflammation [116,117]. Using rat cortical neurons, Wang et al. [118] provided additional evidence that stearic acid protects against lipid peroxidation, an indicator of oxidative stress, via activation of peroxisome proliferator-activated receptor γ. Overall, research demonstrates that dietary stearic acid has dynamic biological actions that may be beneficial for whole body energy metabolism.

### 5.4. Odd- and Branched-Chain FA

Mechanistic studies that examined the metabolic influence of OCFA consumption are limited, although, in general, OCFA are thought to enhance plasma membrane fluidity [119]. Similarly to OCFA, BCFA enhance fluidity of the lipid bilayer [120,121], potentially contributing to beneficial downstream consequences on cellular signaling and transport. In vitro studies have demonstrated *iso* 15:0 to have anti-tumorigenic properties [122,123], and *iso* 17:0 improved β-cell function [124]. In neonatal rats, dietary enrichment with a BCFA mixture (*iso* 14:0, *anteiso* 15:0, *iso* 16:0, *anteiso* 17:0, *iso* 18:0, and *iso* 20:0) reduced the incidence of necrotizing enterocolitis, induced shifts in cecal bacterial composition, and increased expression of anti-inflammatory IL-10 [125]. Yan et al. [126] challenged Caco-2 cells with LPS and observed that treatment with individual BCFA (*iso* 18:0, *iso* 20:0, *anteiso* 13:0, *anteiso* 15:0, or *anteiso* 17:0) attenuated transcription of the inflammatory mediators IL-8 and NF-κB. Similar findings were observed when LPS-stimulated Caco-2 cells treated with a mixture of BCFA that was isolated from the vernix of 20 newborns [127]. Drawing from the limited research available, evidence indicates that BCFA may beneficially modulate health through attenuation of inflammatory responses.

### 5.5. Limitations of Mechanistic Studies Examining Dairy-Derived SFA Consumption on MetS Components

Single nutrient studies are a practical and valuable means to ensure that observed results are unequivocally induced by the dietary component of interest. While this is an appealing advantage compared to studies that utilize whole foods, such a study design possesses its own set of considerable challenges. Most notably, these studies cannot compensate for the fact that humans do not eat dietary FA in isolation. The food matrix, in essence the entire structure and composition of nutrients consumed by an individual, is gaining scientific recognition for its role in modulating the properties and metabolism of any single nutrient it contains [15,16]. Specifically, consumption of isolated dairy-derived nutrients has been found to impact the risk of cardiometabolic disease differently compared to a whole dairy matrix [33]. Dairy products considerably differ in the complexity of their food matrix due to processing methods (e.g*.,* milk vs. cheese), which alters their nutrient types and composition, as well as physical structure [33]. Moreover, studies have shown that differences in food matrix, even within types of dairy products, can modify cardiometabolic risk [128]. Another important consideration is that studies focused on the effects of the consumption of single nutrients often evaluate a very high amount of the nutrient of interest (e.g., 4 g/d butyrate [34,35]). Future research should carefully consider the concentration of a single nutrient that may be attained in an average, balanced diet to enhance the study design’s applicability.

## 6. Conclusions

The broad scope of this narrative review provides a concise glimpse of the current evidence available on the relationship of dairy-derived SFA and MetS risk. A key strength of this review is that it assesses relevant epidemiological and clinical research, as well as mechanistic research from cell culture, animals, and human populations. Recent epidemiological and clinical studies on this subject indicate that intake of dairy-derived SFA is either protective or neutral on cardiometabolic health. Additionally, mechanistic research to date demonstrates that SFA constituents found in dairy fat have additional biochemical functions beyond substrate oxidation, which can have specific and potent consequences on systemic metabolism.

Another strength of this review is the discussion of the evolution of international dietary guidelines, and in particular the alignment of these guidelines with current scientific evidence. This review supports that SFA are a heterogenous class of FA that can vary significantly in their mode of action based their length and structure. However, a lack of relevant controlled clinical trials and mechanistic studies hinders the formation of a clear relationship between dairy-derived SFA consumption and cardiometabolic health. Hence, this review calls into question whether or not dietary guidelines, which discourage the intake of full-fat dairy on the basis of its SFA content, are appropriate. Importantly, this is a narrative review and, as such, the research presented here is not fully comprehensive of all work performed in this field of study. Nevertheless, it is evident that additional research is needed to understand the complex interaction between SFA, food matrix, and disease risk. Future controlled mechanistic and well-designed RCT that consider the complexity of the dairy matrix are needed to clarify the role of dairy-derived SFA intake on MetS.

## Figures and Tables

**Figure 2:**
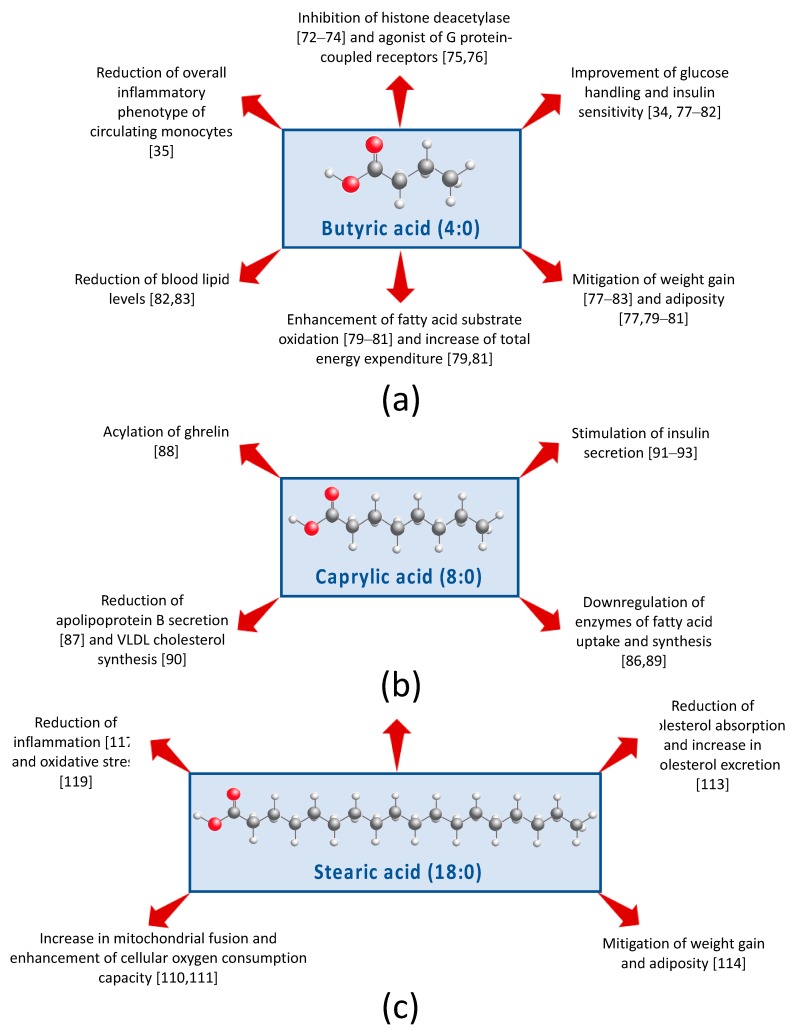
Graphical summary of the potential mechanisms of (**a**) butyric acid, (**b**) caprylic acid, and (**c**) stearic acid that beneficially modulate risk of metabolic syndrome.

**Table 1:** Typical composition (g/100 g) and content (mg/three daily servings) of key saturated fatty acids in bovine-derived milk. Fatty acids are median values in milk, pre-averaged by breed at four different timepoints across a lactation [13].

Fatty acid	Median (g/100 g)	Range (g/100 g)	Median (mg/Three Daily Servings) ^8^
Total SCFA ^1^	5.18	(4.83–7.44)	1150
4:0	3.05	(2.90–5.37)	677
6:0	2.01	(1.86–2.25)	445
Total MCFA ^2^	8.34	(4.03–9.77)	1850
8:0	1.18	(0.82–1.37)	261
10:0	2.86	(1.48–3.38)	635
12:0	3.60	(1.53–4.20)	798
Total LCFA ^3^	58.13	(43.02–67.66)	12,903
14:0	12.14	(8.56–12.57)	2695
16:0	34.83	(25.01–36.60)	7730
18:0	9.05	(7.91–15.94)	2008
Total VLCFA ^4^	0.13	(0.08–0.14)	29
Total OCFA ^5^	2.8	(1.83–2.91)	622
15:0	1.28	(0.72–1.39)	284
17:0	0.71	(0.70–0.97)	158
Total BCFA ^6^	1.65	(1.49–1.8)	366
Total SFA ^7^	72.81	(62.88–76.73)	16,162

^1^ Total SCFA: all short-chain fatty acids (4:0, 5:0, 6:0). ^2^ Total MCFA: all medium-chain fatty acids (7:0, 8:0, 9:0, 10:0, 11:0, cyclohexyl 11:0, 12:0, 13:0). ^3^ Total LCFA: all long-chain fatty acids (14:0, 15:0, 16:0, 17:0, 18:0, 20:0, 21:0). ^4^ Total VLCFA: all very long-chain fatty acids (22:0, 23:0, 24:0). ^5^ Total OCFA: all odd-chain fatty acids (5:0, 7:0, 9:0,11:0, cyclohexyl 11:0, 13:0, 15:0, 17:0, 21:0, 23:0). ^6^ Total BCFA: all branched-chain fatty acids (*iso* 13:0, *anteiso* 13:0, *iso* 14:0, *iso* 15:0, *anteiso* 15:0, *iso* 16:0, *iso* 17:0, *anteiso* 17:0, *iso* 18:0). ^7^ Total SFA: all saturated fatty acids (SCFA, MCFA, LCFA, VLCFA, BCFA). ^8^ Median (mg/three daily servings): Median fatty acids mg/serving of whole milk (3.25% milk fat), calculated from median FA g/100 g as described in Bainbridge et al. (2017) [26] and multiplied by three.

**Table 2:** Summary of observational evidence on the effect of the intake of dairy-derived saturated fatty acids on metabolic syndrome risk.

Reference	Population	Study Design	Adjustments	Risk Factors	
				Hyperglycemia	Metabolic Syndrome (Risk or Prevalence)
Drehmer et al. (2015) [44]	Brazilian adults (10,010; 35–74 year old)	Cross-sectional	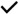	↓	NA ^1^
Drehmer et al. (2016) [21]	Brazilian adults (*n* = 9835; 35–74 year old);	Cross-sectional	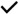	NA	↓
Iggman et al. (2010) [42]	795 Swedish men (~71 year old)	Cross-sectional	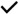	↔ ^2^	NA
Kratz et al. (2014) [45]	American adults with non-alcoholic fatty liver disease (*n* = 17) and controls (*n* = 15)	Cross-sectional	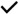	↔	NA
Mayneris-Perxachs et al. (2014) [36]	Spanish adults, asymptomatic with cardiovascular disease risk (*n* = 427; 55–80 year old)	Cross-sectional	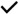	NA	↔
Santaren et al. (2014) [41]	Hispanic, African American, and non-Hispanic white American adults, free of type 2 diabetes at baseline (*n* = 659; 40–60 year old)	Cross-sectional	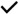	↓	NA
Wanders et al. (2017) [43]	Dutch adults, overweight (*n* = 5675; 45–65 year old)	Cross-sectional	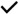	↔	NA

^1^ Parameter not measured or reported. ^2^ Results from parameters measured were a combination of results indicative of increased, decreased, and/or neutral risk.

**Table 3:** Summary of clinical evidence on the effect of the intake of dairy-derived saturated fatty acids on metabolic syndrome risk.

Reference	Population	Study Design	Study Duration	Adjustments	Risk Factors			
					Hyperglycemia	Obesity	Dyslipidemia	Hypertension
Amer et al. (2017) [62], Bohl et al. (2015) [47], Bohl et al. (2017) [46], Matualatupauw et al. (2017) [58]	Danish adults, abdominally overweight (*n* = 52; ≥18 year old)	Parallel	12 weeks		- ^1^	↔ ^2^	-	-
Bjermo et al. (2012) [50]	Swedish adults, abdominally overweight (*n* = 61; 30–65 year old)	Parallel	10 weeks		-	↔	↔	NA ^3^
Iggman et al. (2011) [51]	Swedish adults, hyperlipidemic (*n* = 20; 25–68 year old)	Crossover	9 weeks	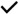	-	-	↔	NA
Intorre et al. (2011) [60]	Italian adults (*n* = 60; 20–40 year old)	Crossover	16 weeks		NA	NA	-	NA
Malpuech-Brugere et al. (2010) [61]	French adults (*n* = 111; 18–50 year old)	Parallel	4 weeks		NA	NA	↔	NA
Pintus et al. (2013) [57]	Italian adults, hypercholesterolemic (*n* = 42; 30–60 year old)	Crossover	9 weeks		NA	-	↔	NA
Venkatramanan et al. (2010) [56]	Canadian adults, overweight and borderline hyperlipidemic (*n* = 15; 30–60 year old)	Crossover	32 weeks		NA	-	-	NA
Wennersberg et al. (2009) [49]	Finnish, Norwegian, and Swedish adult men and postmenopausal women, overweight with traits of MetS (*n* = 121; 30–65 year old)	Parallel	6 months		↔	-	↔	-
Werner et al. (2013) [48]	Danish adult and elderly subjects (*n* = 38; 50–70 year old)	Parallel	12 weeks		-	-	-	NA

^1^ No differences in parameters measured among treatments. ^2^ Parameters measured were a combination of results indicative of increased, decreased, and/or neutral risk. ^3^ Parameter not measured or reported.

## Supplementary Materials

The following is available online at https://www.mdpi.com/2072-6643/11/9/2200/s1, Table S1: Summary of clinical and observational evidence on the effect of the intake of dairy-derived saturated fatty acids on metabolic syndrome risk.

## References

[1] Metabolic Syndrome—Diagnosis and Treatment. https://www.mayoclinic.org/diseases-conditions/metabolic-syndrome/diagnosis-treatment/drc-20351921.

[2] Metabolic Syndrome—National Heart, Lung, and Blood Institute (NHLBI). https://www.nhlbi.nih.gov/health-topics/metabolic-syndrome.

[3] IDF Consensus Worldwide Definition of the Metabolic Syndrome. https://www.idf.org/e-library/consensus-statements/60-idfconsensus-worldwide-definitionof-the-metabolic-syndrome.

[4] Devers M.C., Campbell S., Simmons D. (2016). Influence of age on the prevalence and components of the metabolic syndrome and the association with cardiovascular disease. BMJ Open Diabetes Res. Care.

[5] Moore J.X., Chaudhary N., Akinyemiju T. (2017). Metabolic syndrome prevalence by race/ethnicity and sex in the United States, National Health and Nutrition Examination Survey, 1988–2012. Prev. Chronic Dis..

[6] Dietary Guidelines for Americans 2015–2020. https://health.gov/dietaryguidelines/2015/.

[7] The American Heart Association’s Diet and Lifestyle Recommendations. http://www.heart.org/HEARTORG/HealthyLiving/HealthyEating/Nutrition/The-American-Heart-Associations-Diet-and-Lifestyle-eecommendations_UCM_305855_Article.jsp#.XKItpetKjOQ.

[8] Healthy Diet—World Health Organization. https://www.who.int/news-room/fact-sheets/detail/healthy-diet.

[9] Health Canada Canada’s Dietary Guidelines for Health Professionals and Policy Makers. https://food-guide.canada.ca/en/guidelines/.

[10] Keys A., Anderson J.T., Grande F. (1965). Serum cholesterol response to changes in the diet: IV. Particular saturated fatty acids in the diet. Metabolism.

[11] Houston M. (2018). The relationship of saturated fats and coronary heart disease: Fa(c)t or fiction? A commentary. Ther. Adv. Cardiovasc. Dis..

[12] Zhuang P., Cheng L., Wang J., Zhang Y., Jiao J. (2019). Saturated fatty acid intake is associated with total mortality in a nationwide cohort study. J. Nutr..

[13] Bainbridge M.L., Cersosimo L.M., Wright A.-D. G., Kraft J. (2016). Content and composition of branched-chain fatty acids in bovine milk are affected by lactation stage and breed of dairy cow. PLoS ONE.

[14] Dubois V., Breton S., Linder M., Fanni J., Parmentier M. (2007). Fatty acid profiles of 80 vegetable oils with regard to their nutritional potential. Eur. J. Lipid Sci. Technol..

[15] Guo Q., Ye A., Bellissimo N., Singh H., Rousseau D. (2017). Modulating fat digestion through food structure design. Prog. Lipid Res..

[16] Aguilera J.M. (2018). The food matrix: Implications in processing, nutrition and health. Crit. Rev. Food Sci. Nutr..

[17] Månsson H.L. (2008). Fatty acids in bovine milk fat. Food Nutr. Res..

[18] Kim Y., Je Y. (2016). Dairy consumption and risk of metabolic syndrome: A meta-analysis. Diabet. Med..

[19] Rice B.H. (2014). Dairy and cardiovascular disease: A review of recent observational research. Curr. Nutr. Rep..

[20] Babio N., Becerra-Tomás N., Martínez-González M.Á., Corella D., Estruch R., Ros E., Sayón-Orea C., Fitó M., Serra-Majem L., Arós F. (2015). PREDIMED Investigators Consumption of yogurt, low-fat milk, and other low-fat dairy products is associated with lower risk of metabolic syndrome incidence in an elderly mediterranean population. J. Nutr..

[21] Drehmer M., Pereira M.A., Schmidt M.I., Alvim S., Lotufo P.A., Luft V.C., Duncan B.B. (2016). Total and full-fat, but not low-fat, dairy product intakes are inversely associated with metabolic syndrome in adults. J. Nutr..

[22] Parodi P. (2004). Milk fat in human nutrition. Aust. J. Dairy Technol..

[23] Stergiadis S., Leifert C., Seal C.J., Eyre Larsen M.K., Nielsen J.H., Slots T., Butler G. (2015). A 2-Year study on milk quality of three pasture-based dairy systems of contrasting production intensities in Wales. J. Agric. Sci..

[24] O’Donnell-Megaro A.M., Barbano D.M., Bauman D.E. (2011). Survey of the fatty acid composition of retail milk in the United States including regional and seasonal variations. J. Dairy Sci..

[25] Food Composition Databases Show Foods—Whole Milk, UPC: 070852993669. https://ndb.nal.usda.gov/ndb/foods/show/45339816?fgcd=&manu=none&format=&count=&max=25&offset=&sort=default&order=asc&qlookup=whole+milk&ds=&qt=&qp=&qa=&qn=&q=&ing=.

[26] Bainbridge M.L., Egolf E., Barlow J.W., Alvez J.P., Roman J., Kraft J. (2017). Milk from cows grazing on cool-season pastures provides an enhanced profile of bioactive fatty acids compared to those grazed on a monoculture of pearl millet. Food Chem..

[27] Harfoot C., Hazelwood G., Hobson P., Stewart C. (1997). Lipid metabolism in the rumen. The Rumen Microbial Ecosystem.

[28] Jensen R.G. (2002). The composition of bovine milk lipids: January 1995 to December 2000. J. Dairy Sci..

[29] Wolk A., Vessby B., Ljung H., Barrefors P. (1998). Evaluation of a biological marker of dairy fat intake. Am. J. Clin. Nutr..

[30] Risérus U., Marklund M. (2016). Milk fat biomarkers and cardiometabolic disease. Curr. Opin. Lipidol..

[31] Page I.H., Allen E.V., Chamberlain F.L., Keys A., Stamler J., Stare F.J. (1961). Dietary fat and its relation to heart attacks and strokes. Circulation.

[32] Dietary Goals for the United States. https://thescienceofnutrition.files.wordpress.com/2014/03/dietary-goals-for-the-united-states.pdf.

[33] Thorning T.K., Bertram H.C., Bonjour J.-P., de Groot L., Dupont D., Feeney E., Ipsen R., Lecerf J.M., Mackie A., McKinley M.C. (2017). Whole dairy matrix or single nutrients in assessment of health effects: Current evidence and knowledge gaps. Am. J. Clin. Nutr..

[34] Bouter K., Bakker G.J., Levin E., Hartstra A.V., Kootte R.S., Udayappan S.D., Katiraei S., Bahler L., Gilijamse P.W., Tremaroli V. (2018). Differential metabolic effects of oral butyrate treatment in lean versus metabolic syndrome subjects. Clin. Transl. Gastroenterol..

[35] Cleophas M.C.P., Ratter J.M., Bekkering S., Quintin J., Schraa K., Stroes E.S., Netea M.G., Joosten L.A.B. (2019). Effects of oral butyrate supplementation on inflammatory potential of circulating peripheral blood mononuclear cells in healthy and obese males. Sci. Rep..

[36] Mayneris-Perxachs J., Guerendiain M., Castellote A.I., Estruch R., Covas M.I., Fitó M., Salas-Salvadó J., Martínez-González M.A., Aros F., Lamuela-Raventós R.M. (2014). Plasma fatty acid composition, estimated desaturase activities, and their relation with the metabolic syndrome in a population at high risk of cardiovascular disease. Clin. Nutr..

[37] Kratz M., Baars T., Guyenet S. (2013). The relationship between high-fat dairy consumption and obesity, cardiovascular, and metabolic disease. Eur. J. Nutr..

[38] Rautiainen S., Wang L., Lee I.-M., Manson J.E., Buring J.E., Sesso H.D. (2016). Dairy consumption in association with weight change and risk of becoming overweight or obese in middle-aged and older women: A prospective cohort study. Am. J. Clin. Nutr..

[39] Reaven G.M. (1988). Banting lecture 1988: Role of insulin resistance in human disease. Diabetes.

[40] Laakso M., Kuusisto J. (2014). Insulin resistance and hyperglycaemia in cardiovascular disease development. Nat. Rev. Endocrinol..

[41] Santaren I.D., Watkins S.M., Liese A.D., Wagenknecht L.E., Rewers M.J., Haffner S.M., Lorenzo C., Hanley A.J. (2014). Serum pentadecanoic acid (15:0), a short-term marker of dairy food intake, is inversely associated with incident type 2 diabetes and its underlying disorders. Am. J. Clin. Nutr..

[42] Iggman D., Ärnlöv J., Vessby B., Cederholm T., Sjögren P., Risérus U. (2010). Adipose tissue fatty acids and insulin sensitivity in elderly men. Diabetologia.

[43] Wanders A.J., Alssema M., de Koning E.J.P., le Cessie S., de Vries J.H., Zock P.L., Rosendaal F.R., den Heijer M., de Mutsert R. (2017). Fatty acid intake and its dietary sources in relation with markers of type 2 diabetes risk: The NEO study. Eur. J. Clin. Nutr..

[44] Drehmer M., Pereira M.A., Schmidt M.I., Molina M.D.C.B., Alvim S., Lotufo P.A., Duncan B.B. (2015). Associations of dairy intake with glycemia and insulinemia, independent of obesity, in Brazilian adults: The Brazilian Longitudinal Study of Adult Health (ELSA-Brasil). Am. J. Clin. Nutr..

[45] Kratz M., Marcovina S., Nelson J.E., Yeh M.M., Kowdley K.V., Callahan H.S., Song X., Di C., Utzschneider K.M. (2014). Dairy fat intake is associated with glucose tolerance, hepatic and systemic insulin sensitivity, and liver fat but not β-cell function in humans. Am. J. Clin. Nutr..

[46] Bohl M., Bjørnshave A., Larsen M.K., Gregersen S., Hermansen K. (2017). The effects of proteins and medium-chain fatty acids from milk on body composition, insulin sensitivity and blood pressure in abdominally obese adults. Eur. J. Clin. Nutr..

[47] Bohl M., Bjørnshave A., Rasmussen K.V., Schioldan A.G., Amer B., Larsen M.K., Dalsgaard T.K., Holst J.J., Herrmann A., O’Neill S. (2015). Dairy proteins, dairy lipids, and postprandial lipemia in persons with abdominal obesity (DairyHealth): A 12-wk, randomized, parallel-controlled, double-blinded, diet intervention study. Am. J. Clin. Nutr..

[48] Werner L.B., Hellgren L.I., Raff M., Jensen S.K., Petersen R.A., Drachmann T., Tholstrup T. (2013). Effects of butter from mountain-pasture grazing cows on risk markers of the metabolic syndrome compared with conventional Danish butter: A randomized controlled study. Lipids Health Dis..

[49] Wennersberg M.H., Smedman A., Turpeinen A.M., Retterstøl K., Tengblad S., Lipre E., Aro A., Mutanen P., Seljeflot I., Basu S. (2009). Dairy products and metabolic effects in overweight men and women: Results from a 6-mo intervention study. Am. J. Clin. Nutr..

[50] Bjermo H., Iggman D., Kullberg J., Dahlman I., Johansson L., Persson L., Berglund J., Pulkki K., Basu S., Uusitupa M. (2012). Effects of n-6 PUFAs compared with SFAs on liver fat, lipoproteins, and inflammation in abdominal obesity: A randomized controlled trial. Am. J. Clin. Nutr..

[51] Iggman D., Gustafsson I.-B., Berglund L., Vessby B., Marckmann P., Risérus U. (2011). Replacing dairy fat with rapeseed oil causes rapid improvement of hyperlipidaemia: A randomized controlled study. J. Intern. Med..

[52] Engin A. (2017). The definition and prevalence of obesity and metabolic syndrome. Adv. Exp. Med. Biol..

[53] Kwon H., Kim D., Kim J.S. (2017). Body fat distribution and the risk of incident metabolic syndrome: A longitudinal cohort study. Sci. Rep..

[54] Liu J., Fox C.S., Hickson D.A., May W.D., Hairston K.G., Carr J.J., Taylor H.A. (2010). Impact of abdominal visceral and subcutaneous adipose tissue on cardiometabolic risk factors: The Jackson Heart Study. J. Clin. Endocrinol. Metab..

[55] Kaess B.M., Pedley A., Massaro J.M., Murabito J., Hoffmann U., Fox C.S. (2012). The ratio of visceral to subcutaneous fat, a metric of body fat distribution, is a unique correlate of cardiometabolic risk. Diabetologia.

[56] Venkatramanan S., Joseph S.V., Chouinard P.Y., Jacques H., Farnworth E.R., Jones P.J.H. (2010). Milk enriched with conjugated linoleic acid fails to alter blood lipids or body composition in moderately overweight, borderline hyperlipidemic individuals. J. Am. Coll. Nutr..

[57] Pintus S., Murru E., Carta G., Cordeddu L., Batetta B., Accossu S., Pistis D., Uda S., Elena Ghiani M., Mele M. (2013). Sheep cheese naturally enriched in α-linolenic, conjugated linoleic and vaccenic acids improves the lipid profile and reduces anandamide in the plasma of hypercholesterolaemic subjects. Br. J. Nutr..

[58] Matualatupauw J.C., Bohl M., Gregersen S., Hermansen K., Afman L.A. (2017). Dietary medium-chain saturated fatty acids induce gene expression of energy metabolism-related pathways in adipose tissue of abdominally obese subjects. Int. J. Obes..

[59] Arsenault B.J., Boekholdt S.M., Kastelein J.J.P. (2011). Lipid parameters for measuring risk of cardiovascular disease. Nat. Rev. Cardiol..

[60] Intorre F., Foddai M.S., Azzini E., Martin B., Montel M.-C., Catasta G., Toti E., Finotti E., Palomba L., Venneria E. (2011). Differential effect of cheese fatty acid composition on blood lipid profile and redox status in normolipidemic volunteers: A pilot study. Int. J. Food Sci. Nutr..

[61] Malpuech-Brugère C., Mouriot J., Boue-Vaysse C., Combe N., Peyraud J.-L., LeRuyet P., Chesneau G., Morio B., Chardigny J.-M. (2010). Differential impact of milk fatty acid profiles on cardiovascular risk biomarkers in healthy men and women. Eur. J. Clin. Nutr..

[62] Amer B., Clausen M.R., Bertram H.C., Bohl M., Nebel C., Zheng H., Skov T., Larsen M.K., Gregersen S., Hermansen K. (2017). Consumption of whey in combination with dairy medium-chain fatty acids (MCFAs) may reduce lipid storage due to urinary loss of tricarboxylic acid cycle intermediates and increased rates of MCFAs oxidation. Mol. Nutr. Food Res..

[63] Hodson L., Skeaff C.M., Fielding B.A. (2008). Fatty acid composition of adipose tissue and blood in humans and its use as a biomarker of dietary intake. Prog. Lipid Res..

[64] White P., Chow C.K. (2008). Fatty acids in oilseeds (vegetable oils). Fatty Acids in Foods and Their Health Implications.

[65] Ackman R., Chow C. (2008). Fatty acids in fish and shellfish. Fatty Acids in Foods and Their Health Implications.

[66] Bruckner G., Peng A., Chow C.K. (2008). Vegetables and vegetable products fatty acids. Fatty Acids in Foods and Their Health Implications.

[67] Özogul Y., Özogul F., Çi˙çek E., Polat A., Kuley E. (2009). Fat content and fatty acid compositions of 34 marine water fish species from the Mediterranean Sea. Int. J. Food Sci. Nutr..

[68] Sebedio J.L., Ackman R.G. (1979). Some minor fatty acids of rapeseed oils. J. Am. Oil Chem. Soc..

[69] Jenkins B., Aoun M., Feillet-Coudray C., Coudray C., Ronis M., Koulman A. (2018). The dietary total-fat content affects the in vivo circulating C15:0 and C17:0 fatty acid levels independently. Nutrients.

[70] Ratnayake W.M.N. (2015). Concerns about the use of 15:0, 17:0, and trans-16:1n-7 as biomarkers of dairy fat intake in recent observational studies that suggest beneficial effects of dairy food on incidence of diabetes and stroke. Am. J. Clin. Nutr..

[71] Fellows R., Denizot J., Stellato C., Cuomo A., Jain P., Stoyanova E., Balázsi S., Hajnády Z., Liebert A., Kazakevych J. (2018). Microbiota derived short chain fatty acids promote histone crotonylation in the colon through histone deacetylases. Nat. Commun..

[72] Candido E.P.M., Reeves R., Davie J.R. (1978). Sodium butyrate inhibits histone deacetylation in cultured cells. Cell.

[73] Li H., Gao Z., Zhang J., Ye X., Xu A., Ye J., Jia W. (2012). Sodium butyrate stimulates expression of fibroblast growth factor 21 in liver by inhibition of histone deacetylase 3. Diabetes.

[74] Brown A.J., Goldsworthy S.M., Barnes A.A., Eilert M.M., Tcheang L., Daniels D., Muir A.I., Wigglesworth M.J., Kinghorn I., Fraser N.J. (2003). The Orphan G protein-coupled receptors GPR41 and GPR43 are activated by propionate and other short chain carboxylic acids. J. Biol. Chem..

[75] Stilling R.M., van de Wouw M., Clarke G., Stanton C., Dinan T.G., Cryan J.F. (2016). The neuropharmacology of butyrate: The bread and butter of the microbiota-gut-brain axis?. Neurochem. Int..

[76] Li Z., Yi C.-X., Katiraei S., Kooijman S., Zhou E., Chung C.K., Gao Y., van den Heuvel J.K., Meijer O.C., Berbée J.F.P. (2018). Butyrate reduces appetite and activates brown adipose tissue via the gut-brain neural circuit. Gut.

[77] Lin H.V., Frassetto A., Kowalik E.J., Nawrocki A.R., Lu M.M., Kosinski J.R., Hubert J.A., Szeto D., Yao X., Forrest G. (2012). Butyrate and propionate protect against diet-induced obesity and regulate gut hormones via free fatty acid receptor 3-independent mechanisms. PLoS ONE.

[78] Gao Z., Yin J., Zhang J., Ward R.E., Martin R.J., Lefevre M., Cefalu W.T., Ye J. (2009). Butyrate improves insulin sensitivity and increases energy expenditure in mice. Diabetes.

[79] Henagan T.M., Stefanska B., Fang Z., Navard A.M., Ye J., Lenard N.R., Devarshi P.P. (2015). Sodium butyrate epigenetically modulates high-fat diet-induced skeletal muscle mitochondrial adaptation, obesity and insulin resistance through nucleosome positioning. Br. J. Pharmacol..

[80] Den Besten G., Bleeker A., Gerding A., van Eunen K., Havinga R., van Dijk T.H., Oosterveer M.H., Jonker J.W., Groen A.K., Reijngoud D.-J. (2015). Short-chain fatty acids protect against high-fat diet-induced obesity via a PPARγ-dependent switch from lipogenesis to fat oxidation. Diabetes.

[81] Li H.-P., Chen X., Li M.-Q. (2013). Butyrate alleviates metabolic impairments and protects pancreatic β cell function in pregnant mice with obesity. Int. J. Clin. Exp. Pathol..

[82] Lu Y., Fan C., Li P., Lu Y., Chang X., Qi K. (2016). Short chain fatty acids prevent high-fat-diet-induced obesity in mice by regulating G protein-coupled receptors and gut microbiota. Sci. Rep..

[83] Aguilar E.C., Leonel A.J., Teixeira L.G., Silva A.R., Silva J.F., Pelaez J.M.N., Capettini L.S.A., Lemos V.S., Santos R.A.S., Alvarez-Leite J.I. (2014). Butyrate impairs atherogenesis by reducing plaque inflammation and vulnerability and decreasing NFκB activation. Nutr. Metab. Cardiovasc. Dis..

[84] Luu M., Pautz S., Kohl V., Singh R., Romero R., Lucas S., Hofmann J., Raifer H., Vachharajani N., Carrascosa L.C. (2019). The short-chain fatty acid pentanoate suppresses autoimmunity by modulating the metabolic-epigenetic crosstalk in lymphocytes. Nat. Commun..

[85] Akpa M.M., Point F., Sawadogo S., Radenne A., Mounier C. (2010). Inhibition of insulin and T3-induced fatty acid synthase by hexanoate. Lipids.

[86] Sato K., Cho Y., Tachibana S., Chiba T., Schneider W.J., Akiba Y. (2005). Impairment of VLDL secretion by medium-chain fatty acids in chicken primary hepatocytes is affected by the chain length. J. Nutr..

[87] Kojima M., Hosoda H., Date Y., Nakazato M., Matsuo H., Kangawa K. (1999). Ghrelin is a growth-hormone-releasing acylated peptide from stomach. Nature.

[88] Guo W., Xie W., Han J. (2006). Modulation of adipocyte lipogenesis by octanoate: Involvement of reactive oxygen species. Nutr. Metab. (Lond).

[89] Tachibana S., Sato K., Cho Y., Chiba T., Schneider W.J., Akiba Y. (2005). Octanoate reduces very low-density lipoprotein secretion by decreasing the synthesis of apolipoprotein B in primary cultures of chicken hepatocytes. Biochim. Biophys. Acta Mol. Cell Biol. Lipids.

[90] Stein D.T., Stevenson B.E., Chester M.W., Basit M., Daniels M.B., Turley S.D., McGarry J.D. (1997). The insulinotropic potency of fatty acids is influenced profoundly by their chain length and degree of saturation. J. Clin. Investig..

[91] Leem J., Shim H., Cho H., Park J.-H. (2018). Octanoic acid potentiates glucose-stimulated insulin secretion and expression of glucokinase through the olfactory receptor in pancreatic β-cells. Biochem. Biophys. Res. Commun..

[92] Munakata Y., Yamada T., Imai J., Takahashi K., Tsukita S., Shirai Y., Kodama S., Asai Y., Sugisawa T., Chiba Y. (2018). Olfactory receptors are expressed in pancreatic β-cells and promote glucose-stimulated insulin secretion. Sci. Rep..

[93] Malapaka R.R. V., Khoo S., Zhang J., Choi J.H., Zhou X.E., Xu Y., Gong Y., Li J., Yong E.-L., Chalmers M.J. (2012). Identification and mechanism of 10-carbon fatty acid as modulating ligand of peroxisome proliferator-activated receptors. J. Biol. Chem..

[94] Sengupta A., Ghosh M. (2012). Comparison of native and capric acid-enriched mustard oil effects on oxidative stress and antioxidant protection in rats. Br. J. Nutr..

[95] Wang B., Fu J., Li L., Gong D., Wen X., Yu P., Zeng Z. (2016). Medium-chain fatty acid reduces lipid accumulation by regulating expression of lipid-sensing genes in human liver cells with steatosis. Int. J. Food Sci. Nutr..

[96] Lee S.I., Kang K.S. (2017). Function of capric acid in cyclophosphamide-induced intestinal inflammation, oxidative stress, and barrier function in pigs. Sci. Rep..

[97] Ma J., Checklin H.L., Wishart J.M., Stevens J.E., Jones K.L., Horowitz M., Meyer J.H., Rayner C.K. (2013). A randomised trial of enteric-coated nutrient pellets to stimulate gastrointestinal peptide release and lower glycaemia in type 2 diabetes. Diabetologia.

[98] Alves N.F.B., de Queiroz T.M., de Almeida Travassos R., Magnani M., de Andrade Braga V. (2017). Acute treatment with lauric acid reduces blood pressure and oxidative stress in spontaneously hypertensive rats. Basic Clin. Pharmacol. Toxicol..

[99] Ong M.-H.-L., Wong H.-K., Tengku-Muhammad T.-S., Choo Q.-C., Chew C.-H. (2019). Pro-atherogenic proteoglycanase ADAMTS-1 is down-regulated by lauric acid through PI3K and JNK signaling pathways in THP-1 derived macrophages. Mol. Biol. Rep..

[100] Maurer-Stroh S., Gouda M., Novatchkova M., Schleiffer A., Schneider G., Sirota F.L., Wildpaner M., Hayashi N., Eisenhaber F. (2004). MYRbase: Analysis of genome-wide glycine myristoylation enlarges the functional spectrum of eukaryotic myristoylated proteins. Genome Biol..

[101] Ren X.-M., Cao L.-Y., Zhang J., Qin W.-P., Yang Y., Wan B., Guo L.-H. (2016). Investigation of the binding interaction of fatty acids with human G protein-coupled receptor 40 using a site-specific fluorescence probe by flow cytometry. Biochemistry.

[102] Snook J.T., Park S., Williams G., Tsai Y.-H., Lee N. (1999). Effect of synthetic triglycerides of myristic, palmitic, and stearic acid on serum lipoprotein metabolism. Eur. J. Clin. Nutr..

[103] Temme E.H., Mensink R.P., Hornstra G. (1997). Effects of medium chain fatty acids (MCFA), myristic acid, and oleic acid on serum lipoproteins in healthy subjects. J. Lipid Res..

[104] Takato T., Iwata K., Murakami C., Wada Y., Sakane F. (2017). Chronic administration of myristic acid improves hyperglycaemia in the Nagoya–Shibata–Yasuda mouse model of congenital type 2 diabetes. Diabetologia.

[105] Wada Y., Sakiyama S., Sakai H., Sakane F. (2016). Myristic acid enhances diacylglycerol kinase δ-dependent glucose uptake in myotubes. Lipids.

[106] Iwata K., Sakai H., Takahashi D., Sakane F. (2019). Myristic acid specifically stabilizes diacylglycerol kinase δ protein in C2C12 skeletal muscle cells. Biochim. Biophys. Acta Mol. Cell Biol. Lipids.

[107] Rioux V., Catheline D., Beauchamp E., Le Bloc’h J., Pédrono F., Legrand P. (2008). Substitution of dietary oleic acid for myristic acid increases the tissue storage of α-linolenic acid and the concentration of docosahexaenoic acid in the brain, red blood cells and plasma in the rat. Animal.

[108] Rioux V., Catheline D., Bouriel M., Legrand P. (2005). Dietary myristic acid at physiologically relevant levels increases the tissue content of C20:5 n-3 and C20:3 n-6 in the rat. Reprod. Nutr. Dev..

[109] Senyilmaz D., Virtue S., Xu X., Tan C.Y., Griffin J.L., Miller A.K., Vidal-Puig A., Teleman A.A. (2015). Regulation of mitochondrial morphology and function by stearoylation of TFR1. Nature.

[110] Senyilmaz-Tiebe D., Pfaff D.H., Virtue S., Schwarz K.V., Fleming T., Altamura S., Muckenthaler M.U., Okun J.G., Vidal-Puig A., Nawroth P. (2018). Dietary stearic acid regulates mitochondria in vivo in humans. Nat. Commun..

[111] Hassel C.A., Mensing E.A., Gallaher D.D. (1997). Dietary stearic acid reduces plasma and hepatic cholesterol concentrations without increasing bile acid excretion in cholesterol-fed hamsters. J. Nutr..

[112] Schneider C.L., Cowles R.L., Stuefer-Powell C.L., Carr T.P. (2000). Dietary stearic acid reduces cholesterol absorption and increases endogenous cholesterol excretion in hamsters fed cereal-based diets. J. Nutr..

[113] Gouk S.-W., Cheng S.-F., Soon-Hock Ong A., Chuah C.-H. (2013). Stearic acids at sn-1, 3 positions of TAG are more efficient at limiting fat deposition than palmitic and oleic acids in C57BL/6 mice. Br. J. Nutr..

[114] Hunter J.E., Zhang J., Kris-Etherton P.M. (2010). Cardiovascular disease risk of dietary stearic acid compared with trans, other saturated, and unsaturated fatty acids: A systematic review. Am. J. Clin. Nutr..

[115] Cowles R.L., Lee J.-Y., Gallaher D.D., Stuefer-Powell C.L., Carr T.P. (2002). Dietary stearic acid alters gallbladder bile acid composition in hamsters fed cereal-based diets. J. Nutr..

[116] Nishitani Y., Okazaki S., Imabayashi K., Katada R., Umetani K., Yajima H., Matsumoto H. (2007). Saturated and monounsaturated fatty acids increase interleukin-10 production in rat hepatocytes. Nihon Arukoru Yakubutsu Igakkai Zasshi.

[117] Pan P.-H., Lin S.-Y., Ou Y.-C., Chen W.-Y., Chuang Y.-H., Yen Y.-J., Liao S.-L., Raung S.-L., Chen C.-J. (2010). Stearic acid attenuates cholestasis-induced liver injury. Biochem. Biophys. Res. Commun..

[118] Wang Z., Liang C., Li G., Yu C., Yin M. (2007). Stearic acid protects primary cultured cortical neurons against oxidative stress. Acta Pharmacol. Sin..

[119] Holman R.T., Adams C.E., Nelson R.A., Grater S.J., Jaskiewicz J.A., Johnson S.B., Erdman J.W. (1995). Patients with anorexia nervosa demonstrate deficiencies of selected essential fatty acids, compensatory changes in nonessential fatty acids and decreased fluidity of plasma lipids. J. Nutr..

[120] Kaneda T. (1991). Iso-and anteiso-fatty acids in bacteria: Biosynthesis, function, and taxonomic significance. Microbiol. Rev..

[121] Poger D., Caron B., Mark A.E. (2014). Effect of methyl-branched fatty acids on the structure of lipid bilayers. J. Phys. Chem. B.

[122] Cai Q., Huang H., Qian D., Chen K., Luo J., Tian Y., Lin T., Lin T. (2013). 13-Methyltetradecanoic acid exhibits anti-tumor activity on T-cell lymphomas in vitro and in vivo by down-regulating p-AKT and activating caspase-3. PLoS ONE.

[123] Yang Z., Liu S., Chen X., Chen H., Huang M., Zheng J. (2000). Induction of apoptotic cell death and in vivo growth inhibition of human cancer cells by a saturated branched-chain fatty acid, 13-methyltetradecanoic acid. Cancer Res..

[124] Kraft J., Jetton T., Satish B., Gupta D. (2015). Dairy-derived bioactive fatty acids improve pancreatic ß-cell function. FASEB J..

[125] Ran-Ressler R.R., Khailova L., Arganbright K.M., Adkins-Rieck C.K., Jouni Z.E., Koren O., Ley R.E., Brenna J.T., Dvorak B. (2011). Branched chain fatty acids reduce the incidence of necrotizing enterocolitis and alter gastrointestinal microbial ecology in a neonatal rat model. PLoS ONE.

[126] Yan Y., Wang Z., Greenwald J., Kothapalli K.S.D., Park H.G., Liu R., Mendralla E., Lawrence P., Wang X., Brenna J.T. (2017). BCFA suppresses LPS induced IL-8 mRNA expression in human intestinal epithelial cells. Prostaglandins Leukot. Essent. Fat. Acids.

[127] Yan Y., Wang Z., Wang D., Lawrence P., Wang X., Kothapalli K.S.D., Greenwald J., Liu R., Park H.G., Brenna J.T. (2018). BCFA-enriched vernix-monoacylglycerol reduces LPS-induced inflammatory markers in human enterocytes in vitro. Pediatr. Res..

[128] Mozaffarian D., Hao T., Rimm E.B., Willett W.C., Hu F.B. (2011). Changes in diet and lifestyle and long-term weight gain in women and men. N. Engl. J. Med..

