# Supplementary material for: Dairy Fat Consumption and the Risk of Metabolic Syndrome: An Examination of the Saturated Fatty Acids in Dairy
Source: Nutrients. 2019 Sep 12;11(9):2200. doi: 10.3390/nu11092200 (PMC6769731; doi:10.3390/nu11092200)
Supplement: Supplementary file 1 [file nutrients-11-02200-s001.pdf]

Table S1. Summary of clinical and observational evidence on the effect of the intake of dairy-derived saturated fatty acids on metabolic syndrome risk.

Reference	Study objective	Population	Study design	Study duration	Adjustments	Results
			Randomized, controlled trials			
Amer et al. (2017) [50]	Investigate the influence of milk lipids with high or low content of medium-chain fatty acids and milk protein consumption on the blood and urine metabolic profiles	Danish adults, abdominally overweight (n = 52; ≥ 18 year old)	Parallel	12 weeks	-	High vs. low medium-chain SFA butter diet: • ↑ urinary levels of adipic acid
Bjermo et al. (2012) [39]	Investigate the effects of SFA or PUFA-rich diet on liver fat, systemic inflammation, and metabolic disorders	Swedish adults, abdominally overweight (n = 61; 30-65 year old); Role of Dietary Fatty Acids in Fatty Liver and Insulin Resistance (HEPFAT) trial	Parallel	10 weeks	-	SFA vs. PUFA diet: • No difference in glucose measurements • No difference in body weight, BMI, waist circumference, percent body fat or adipose tissue gene expression • ↑ visceral/subcutaneous adipose tissue ratio • No difference in plasma triacylglycerols, HDL cholesterol, or LDL cholesterol • ↑ plasma cholesterol
Bohl et al. (2015) [36]	Investigate whether dairy-derived protein and medium-chain SFA	Danish adults, abdominally overweight (n = 52; ≥ 18 year old)	Parallel	12 weeks	-	High vs. low medium-chain SFA butter diet: • No difference in glucose measurements

	supplementation improves postprandial lipid metabolism					• No difference in body weight and adipose tissue gene expression<br>• No difference in blood lipids
Bohl et al. (2017) [35]	Investigate whether intake of whey protein and butter naturally enriched in medium-chain SFA affects body composition, insulin sensitivity, blood pressure and plasma cholesterol concentrations	Danish adults, abdominally overweight (*n* = 52; ≥ 18 year old)	Parallel	12 weeks	-	• High vs. low medium-chain SFA butter diet:<br>• ↑ lean body mass and ↓ total body and gynoid fat percentage<br>• No difference in glucose homeostasis<br>• No difference in blood lipids<br>• No difference in blood pressure
Iggman et al. (2011) [40]	Investigate effect of replacing dietary dairy fat or rapeseed oil on blood lipids, glucose metabolism, and coagulation factors	Adult Swedish subjects, hyperlipidemic (*n* = 20; 25–68 year old)	Crossover	9 weeks	Body weight	Dairy fat vs. rapeseed oil diet:<br>• ↑ total cholesterol, LDL cholesterol, total/HDL cholesterol ratio, and triacylglycerols<br>• No difference in HDL cholesterol, insulin sensitivity, fasting glucose, and glucose tolerance
Intorre et al. (2011) [48]	Investigate effect of intake of two cheese varieties differing in fat quality on blood lipids and redox status	Italian adults (*n* = 60; 20–40 year old)	Crossover	16 wk	-	Control vs. experimental diet:<br>• No difference in plasma total cholesterol, HDL cholesterol, LDL cholesterol and triacylglycerols

Malpuech-Brugere et al. (2010) [49]	Investigate impact on CVD risk factors of milk fats with varying ratios between *trans* fatty acids, SFA, MUFA[13], and PUFA	French adults (*n* = 111; 18–50 year old)	Parallel	4 weeks	-	72%, 63%, or 57% SFA milk fat diet:<br>• No difference in HDL cholesterol.<br>• ↓ total cholesterol, LDL cholesterol, and LDL/HDL cholesterol ratio in 63% SFA milk fat diet compared to 72% diet and ↓ total/HDL cholesterol ratio compared to 72% and 57% milk fat diet
Matualatupauw et al. (2017) [47]	Investigate effects of high medium-chain SFA vs. low medium-chain SFA diet on subcutaneous adipose tissue gene expression	Danish adults, abdominally overweight (*n* = 52; ≥ 18 year old)	Parallel	12 weeks	-	High vs. low medium-chain SFA butter diet:<br>• ↑ expression of gene sets related to citric acid cycle, oxidative phosphorylation, and adipogenesis<br>• PPARα and PPAR γ coactivator 1-α were predicted to be activated by medium-chain SFA
Pintus et al. (2013) [46]	Investigate effects of sheep cheese enriched in α-linolenic, conjugated linoleic, and vaccenic acids on plasma lipid and endocannabinoid profiles	Italian adults, hypercholesterolemic (*n* = 42; 30–60 year old)	Crossover	9 weeks	-	59% vs. 46% SFA cheese diet:<br>• No difference when consuming 45 or 90 g/d of cheese on BMI or waist circumference<br>• No difference when consuming 45 g/d of cheese on blood lipids

						• ↑ total cholesterol, HDL cholesterol, and LDL cholesterol when consuming 90 g/d
Venkatramanan et al. (2010) [45]	Investigate whether consumption of milk enriched with 9*c*,11*t* and 10*t*,12*c* 18:2 isomers alters blood lipids; liver function; C-reactive protein; tumor necrosis factor-alpha; and body weight and composition	Canadian adults, overweight and borderline hyperlipidemic (*n* = 15; 30–60 year old)	Crossover	32 weeks	-	60% vs. 48% SFA milk diet: • No difference in body weight or composition • No difference in triacylglycerols, total cholesterol, LDL cholesterol, or HDL cholesterol
Wennersberg et al. (2009) [38]	Investigate whether increased intake of dairy products in subjects who habitually consumed a low amount of dairy beneficially affects abdominal obesity, and/or other factors of MetS	Finnish, Norwegian, and Swedish adult men and postmenopausal women, overweight with traits of MetS (*n* = 121; 30–65 year old)	Parallel	6 months	-	Milk vs. control diet: • No difference in glucose homeostasis measurements except ↑ insulin sensitivity • No difference in body weight, BMI, waist circumference, body fat mass and proportion • No difference in blood lipids except ↑ total cholesterol
Werner et al. (2013) [37]	Investigate effects of intake of butter from mountain-pasture grazing cows compared to	Danish adult and elderly (*n* = 38; 50–70 year old)	Parallel	12 weeks	-	59% vs. 64% SFA butter diet: • No difference in insulin, plasma glucose, or glucose tolerance

	butter from conventionally-produced cows on risk markers of cardiovascular disease and type 2 diabetes					• No difference in body weight<br>• No difference in serum total cholesterol, LDL cholesterol, HDL cholesterol, or triacylglycerols.
Observational studies						
Drehmer et al. (2015) [33]	Investigate association between consumption of dairy products and glycemic status	Brazilian adults (10,010; 35–74 year old); Brazilian Longitudinal Study of Adult Health (ELSA-Brasil)	Cross-sectional	-	Age, sex, race, occupational status, education, family income, study center, menopause, family history of diabetes, smoking status, alcohol intake, physical activity, calorie intake, and non-dairy foods, height, waist, and hip measurements	• Inverse association between dairy intake and fasting glucose, 2 hr post-load glucose, and hemoglobin A1c was attenuated when myristic acid was included as an adjustment to the model.
Drehmer et al. (2016) [5]	Investigate association of dairy, type of dairy, and dairy fat intake on MetS	Brazilian adults and elderly (*n* = 9,835; 35–74 year old); Brazilian Longitudinal Study of Adult Health (ELSA-Brasil)	Cross-sectional	-	Age, sex, race, occupational status, education, family income, study center, menopausal status, family history of	• Inverse association between total and full-fat dairy intake with MetScore was attenuated when dairy-derived SFA intake was included as an adjustment to the model.

					diabetes, smoking status, alcohol intake, physical activity, and nondairy variables	
Iggman et al. (2010) [31]	Investigate association between individual adipose tissue fatty acids and insulin sensitivity	795 Swedish men (~71 yr old); Uppsala Longitudinal Study of Adult Men (ULSAM)	Cross-sectional	-	BMI, smoking, alcohol intake, and physical activity	• 17:0 (% of total fatty acids) of adipose tissue biopsies was positively correlated with insulin sensitivity
Kratz et al. (2014) [34]	Investigate association of intake of *trans*-16:1 n-7 and intake of dairy fat with glucose tolerance, insulin sensitivity, and β-cell function	American adults with NAFLD (*n* = 17) with age- and BMI-matched controls (*n* = 15)	Cross-sectional	-	Age, sex, BMI, liver-spleen ratio	• Plasma phospholipid 15:0 and 17:0 (% of total phospholipid) of was negatively associated with fasting glucose<br>• Phospholipid 17:0 and FFA 15:0 (% of total phospholipid and FFA, respectively) was associated with glucose tolerance<br>• No association of plasma phospholipid (% of total phospholipid) or FFA 15:0 and 17:0 (% of total FFA) with insulin resistance or β-cell function
Mayneris-Perxachs et al. (2014) [27]	Analyze plasma fatty acid composition and estimated desaturase	Spanish adults, asymptomatic with high risk of cardiovascular disease (*n* = 427; 55–80 year old); Prevention	Cross-sectional	-	Sex, age, energy intake, BMI, smoking status, occupation, and educational level	• Second quartile of plasma 17:0 (% of total fatty acids) was associated with ↓ risk of MetS compared to

	activities in relation to MetS status; examine associations between these patterns and MetS and its components	with Mediterranean Diet (PREDIMED) study				first quartile, but not third or fourth quartiles
Santaren et al. (2014) [30]	Investigate association of dairy fatty acid biomarkers, 15:0 acid and *trans*-16:1 n-7, with insulin resistance and β-cell function, and with incident type 2 diabetes after 5-yr follow-up	Hispanic, African American, and non-Hispanic white American adults, free of type 2 diabetes at baseline (*n* = 659; 40–60 year old)	Cross-sectional	-	Age, sex, ethnicity, center, physical activity, smoking status, alcohol intake, education, total energy/fruit and vegetable/ red meat/ soft drink/fiber intakes	• 15:0 concentration was positively associated to log S_I (insulin sensitivity index) and log DI (disposition index) • Additional adjustment for BMI and waist circumference attenuated this association
Wanders et al. (2017) [32]	Investigate relationship of intake of total fatty acids, SFA, MUFA, PUFA *and* trans fatty acids with blood markers of diabetes risk; Investigate possible influence dietary sources of FA on these associations	Dutch adults, overweight (*n* = 5675; 45–65 year old); Netherlands Epidemiology of Obesity (NEO) study	Cross-sectional	-	Age, sex, energy intake, alcohol intake, fiber intake, body fat percentage, smoking status, education level, physical activity, family history of diabetes, statin use, energy under-reporter, coffee intake, sugar-sweetened beverage intake; dietary	• Dairy-derived SFA intake was inversely associated with fasting insulin and HOMA-B

			cholesterol,<br>protein intake,<br>FA intake	
